# SARS-CoV-2 Placental Infection in an Unvaccinated Mother Resulting in Fetal Demise

**DOI:** 10.7759/cureus.20833

**Published:** 2021-12-30

**Authors:** Dominic J Bewley, Jessica Lee, Oana Popescu, Angelica Oviedo

**Affiliations:** 1 Pathology, Burrell College of Osteopathic Medicine, Las Cruces, USA; 2 Pathology and Laboratory Medicine, Vernon Jubilee Hospital, Vernon, CAN; 3 Pathology and Laboratory Medicine, Burrell College of Osteopathic Medicine, Las Cruces, USA

**Keywords:** unvaccinated, intervillositis, fetal demise, placenta, sars-cov-2 infection

## Abstract

Severe acute respiratory syndrome coronavirus 2 (SARS-CoV-2) infection in pregnancy may have devastating complications including fetal demise. Here, we describe a case of SARS-CoV-2 infection in a second-trimester pregnancy. The placenta demonstrated the presence of SARS-CoV-2 viral RNA along with intervillositis and perivillous fibrin deposition. SARS-CoV-2 directly infects the trophoblastic cells of the placenta through the angiotensin-converting enzyme 2 receptor. This case report details the clinical history of a SARS-CoV-2-infected pregnancy with fetal demise. In addition, we show the images of the placental pathology and SARS-CoV-2 RNA in situ studies.

## Introduction

Despite being well into the severe acute respiratory syndrome coronavirus 2 (SARS-CoV-2) pandemic, the implications of SARS-CoV-2 in pregnancy are yet to be elucidated. Although there are numerous reports of pregnant women with SARS-CoV-2 infection, the effects on the fetus remain unknown [1]. Additionally, it is unclear whether the trimester when the infection occurs is important for pregnancy complications. Moreover, the role of vaccination in maternal and fetal health is yet to be determined. In addition, maternal deaths due to SARS-CoV-2 infection have been reported [2]. The SARS-CoV-2 vaccine has been approved for use in pregnant women since early 2021 in North America [3]. Despite this, many pregnant women are unvaccinated. Here, we report a case of SARS-CoV-2 infection in an unvaccinated mother that resulted in fetal death.

Previous studies have shown that the maternal-placental interface is a major factor in complications of SARS CoV-2-infected pregnancies. This likely results in maternal pregnancy complications such as intensive care unit admission, mechanical ventilation, extracorporeal membrane oxygenation, and thromboembolic disease. Pregnant women infected with SARS-CoV-2 are twice as likely to die as non-pregnant women [2].

The main receptor for SARS-CoV-2 is angiotensin-converting enzyme 2 (ACE2), which is found mainly in pulmonary alveolar epithelial cells [4]. ACE2 receptor is also found in the trophoblastic cells of the placenta. Placental infection by SARS-CoV-2 occurs through ACE2 receptor-mediated mechanisms [5].

In addition, SARS-CoV-2 infection during pregnancy can cause an increased risk of fetal demise [6]. We report a case of fetal demise in a SARS-CoV-2-infected mother with detailed pathology. Although there are comparable reports in infectious literature, it is important to continue to disseminate this knowledge in general medical literature to encourage unvaccinated individuals to get vaccinated. In addition, precautionary measures and careful monitoring should be conducted among pregnant women who have had SARS-CoV-2 exposure or infection.

The mode of transmission of SARS-CoV-2 from the mother to the infant is important to understand. Possibilities include prenatal transplacental transmission and blood exposure during delivery. Postnatal transmission could occur through breastfeeding or airborne transmission from the mother to the neonate.

During the time of our patient’s infection, there was a regional surge of the Delta variant of SARS-CoV-2, which likely infected our patient.

## Case presentation

A 27-year-old G3P0 woman delivered at 24 weeks and five days which led to fetal demise. The mother’s blood group was A+, she was immune from rubella, and tested negative for human immunodeficiency virus, hepatitis B, and sexually transmitted diseases. Moreover, she was unvaccinated for the SARS-CoV-2. Blood pressure ranged from 130/80 to 140/80 mmHg. The slightly elevated blood pressure was considered not clinically significant. No further workup was done, and no medications were given. This was not considered to be a sign of preeclampsia.

The mother was a known epilepsy patient on lamotrigine and Dilantin. She had a history of generalized tonic-clonic seizures with electroencephalography showing bilateral frontotemporal electrocerebral dysfunction. When the patient became pregnant, Dilantin was discontinued. She had a seizure during the first trimester after the discontinuation of Dilantin. A computed tomography scan performed at the time showed bilateral subcortical calcification of frontal and parietal lobes with no intracranial bleed, while magnetic resonance imaging was suggestive of a vascular malformation.

At 20 weeks of gestation, an anatomy ultrasound was normal. At 24 weeks and two days of gestation, the mother suffered a dog bite that punctured the skin on the forearm. The wound was sutured in the Emergency Department, and she was given amoxicillin/clavulanic acid. The wound improved and there were no further complications. The day after the dog bite, her family physician examined her and noted the fetal heart rate was approximately 130 beats per minute. Later that day, the mother complained of rhinorrhea, fatigue, cough, and fever of 38.8°C which responded to acetaminophen. Her fever persisted for the following 48 hours. Other household members reported similar symptoms. The mother noted the absence of fetal movement two days after symptom onset. Four days after symptom onset, an ultrasound identified fetal demise. During labor induction, the SARS-CoV-2 swab test for the mother was positive.

The mother gave consent for an autopsy of the fetus. Examination showed a normally formed male fetus with growth parameters consistent with 24-25 weeks of gestation. Histological examination of the organs was normal. Polymerase chain reaction for SARS-CoV-2 on the fetal liver was negative. Chromosome microarray demonstrated a normal male complement.

**Figure 1:**
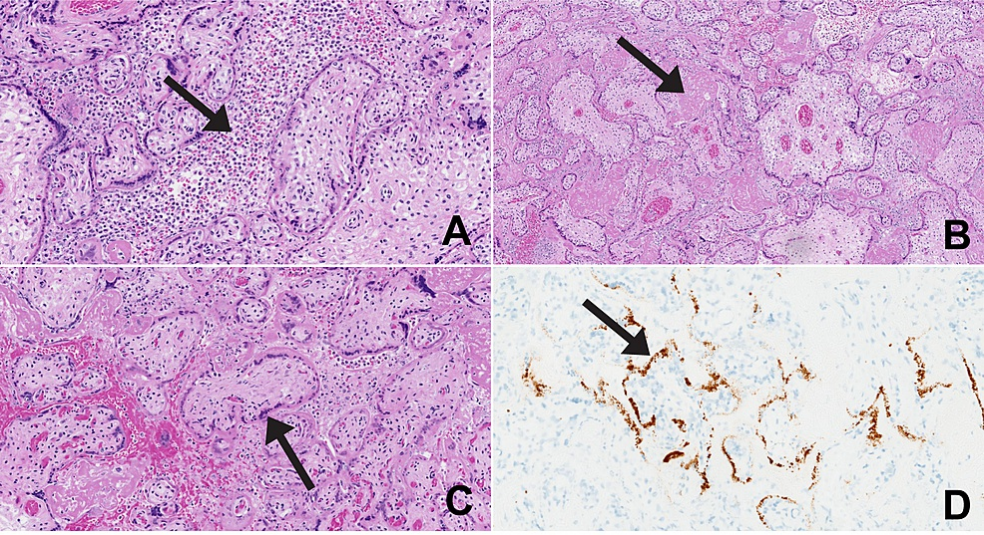
The SARS-CoV-2 infected placenta. A: intervillositis with acute and chronic inflammation in the intervillous space. B: perivillous fibrin deposition secondary to severe inflammation. C: villi with loss of nuclear staining in cytotrophoblast and syncytiotrophoblast. D: SARS-CoV-2 RNA in situ positive in cytotrophoblast and syncytiotrophoblast. SARS-CoV-2: severe acute respiratory syndrome coronavirus 2

The placental disk weighed 215 g (formalin-fixed), the expected weight for 24-25 weeks of gestation is 150-256 g. Gross examination demonstrated a normal umbilical cord and membranes. Cut sections of the placenta showed diffuse beige material that occupied 90% of the parenchyma, suggestive of perivillous fibrin deposition. Microscopic examination showed a placenta consistent with gestational age. The intervillous space was largely filled with inflammatory cells composed of macrophages, neutrophils, and lymphocytes, which was interpreted as acute and chronic intervillositis (Figure 1).

There were also large amounts of perivillous fibrin deposition. There were multiple intervillous thrombi. Most of the villi showed loss of nuclear staining of the cytotrophoblast and syncytiotrophoblast, suggestive of viral cytopathic effect or hypoxic change because of the inflammation. These areas of loss of nuclear staining corresponded to regions of positivity seen in the SARS-CoV-2 RNA in situ sections (SARS-CoV-2 viral RNA using the V-nCoV2019-S probe to detect viral replication with the V-nCoV2019-S sense probe, Advanced Cell Diagnostics, Inc., Newark, CA). The placenta showed no histologic correlates of hypertension.

## Discussion

Studies have reported that the main mode of transmission of SARS-CoV-2 from the mother to the fetus is vertical. Placentitis due to intervillositis has been described as the primary pathology in the SARS-CoV-2-infected placenta. There have been numerous reports of stillbirth due to SARS-CoV-2 intervillositis [7]. SARS-CoV-2 intervillositis has also resulted in both infected and non-infected neonates [6].

Our patient was a known epilepsy patient with focal calcification on brain imaging. Therefore, the seizure during the first trimester was attributed to seizure medication changes and not eclampsia. The seizure history with lamotrigine treatment was considered incidental and not likely contributory to the severe SARS-CoV-2 placental infection.

SARS-CoV-2 is capable of infecting the placenta through the ACE2 receptors located on the trophoblastic cells [5]. Perivillous fibrin deposition is considered secondary to severe infection. Our findings support the mechanism of demise or stillbirth to be severe placental insufficiency due to acute and chronic intervillositis with perivillous fibrin deposition due to SARS-CoV-2 infection.

Although the patient had slightly elevated blood pressure during pregnancy, it was not considered clinically significant and no further workup was done. In addition, pathologic examination of the placenta showed normal weight and no histologic signs of hypertensive disease during pregnancy. The fetus also had normal growth parameters, and there were no signs of chronic uteroplacental insufficiency. This was interpreted as elevated blood pressure having no direct effect on the pregnancy. However, it cannot be determined at this time if the elevated blood pressure contributed to the severe SARS-CoV-2 infection of the placenta. In non-pregnant SARS-CoV-2 populations, hypertension is considered a major risk factor for severe disease [8]. Further investigations of SARS-CoV-2-infected pregnancies and associated blood pressure are needed to answer this critical question.

It is important to understand the implications of SARS-CoV-2 infection in pregnancy. To make treatment-related decisions during pregnancy and prevent complications, there is much to be learned. Although our case report describes a second-trimester demise, additional studies are needed to determine the effect of gestational age on pregnancy outcome. In addition, maternal factors may play a role in the outcome of SARS-CoV-2 infection on fetal health. Vaccination status is highly likely to play a role in pregnancy outcomes. The role of hypertension in SARS-CoV-2-infected pregnancies also requires further investigation.

## Conclusions

We present this case of SARS-CoV-2 infection during pregnancy that resulted in demise and show the associated placental pathology with acute and chronic intervillositis and perivillous fibrin deposition. In addition, we describe the detailed clinical history of this SARS-CoV-2 second-trimester infection. Further investigation is needed to understand the many complicated fetal, viral, and maternal factors, including hypertension, that are important in determining pregnancy outcomes in SARS-CoV-2-infected mothers. We present this case report to add to the medical literature and encourage unvaccinated individuals to get vaccinated. Precautionary measures and additional monitoring should be performed for pregnant women with SARS-CoV-2 infection.
